# Nested mobile genetic elements mediating antimicrobial resistance genes mobility within and between *Acinetobacter* isolates

**DOI:** 10.1016/j.isci.2026.115648

**Published:** 2026-04-08

**Authors:** Kenneth Bongulto, Ngure Kagia, Hisamichi Tauchi, Satoru Suzuki, Kozo Watanabe

**Affiliations:** 1 Center for Marine Environmental Studies (CMES), Ehime University, Bunkyo-cho 3, Matsuyama, Ehime 790-8577, Japan; 2 Graduate School of Science and Engineering, Ehime University, Bunkyo-cho 3, Matsuyama, Ehime 790-8577, Japan; 3 Ehime University Graduate School of Medicine, Toon, Ehime, Japan

**Keywords:** classification description, microbial genomics

## Abstract

Mobile genetic elements (MGEs) play a central role in the acquisition and dissemination of antibiotic resistance genes (ARGs). This study analyzed the distribution of MGEs using the whole-genome sequences of 38 *Acinetobacter* isolates from patient, environmental, and pig waste samples. Pig waste isolates exhibited the highest mean number of plasmids, while prophages were more prevalent in environment-associated isolates. Interestingly, we observed a significant positive correlation between number of plasmids and number of defense systems. Co-localization of multiple ARGs within a single plasmid was observed, with up to 10 distinct ARGs observed within a single p*dif* module. Additionally, putative genomic resistance islands (GRIs) were identified in non-baumannii *Acinetobacter* species, representing the first documentation of GRIs outside the *Acinetobacter calcoaceticus-baumannii* (Acb) complex. This study provides new insights into the mechanisms of ARG dissemination in *Acinetobacter*, particularly the role of MGEs in facilitating hierarchical gene transfer processes.

## Highlights

• Plasmids possessed multiple ARGs and bacterial defense systems
• Antimicrobial resistance genes (ARGs) are enriched in p*dif* modules
• Nested MGEs enabled hierarchical ARG transfer within and between bacterial cells
• Putative genomic resistance islands (GRIs) were observed in non-*baumannii* species

## Graphical abstract

**Figure:**
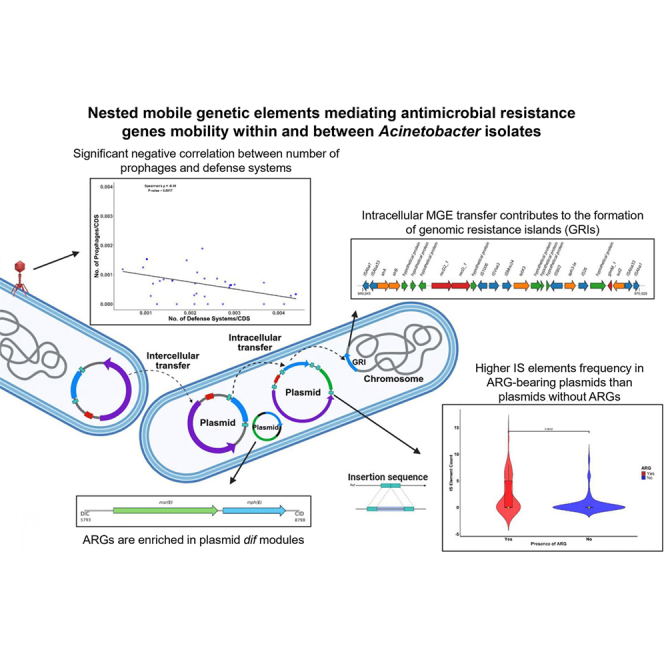

## Introduction

Horizontal gene transfer (HGT) is a fundamental driver of bacterial evolution, enabling the rapid acquisition of beneficial genetic traits that enhance fitness and survival.^1^ Through various mechanisms including transformation, conjugation, transduction, and vesiduction, bacteria can acquire diverse functional genes.^2^ Mobile genetic elements (MGEs) serve as key mediators of these processes, facilitating gene transfer both within and between cells.^3^^,^^4^ These gene transfers are significant for bacterial adaptation, particularly in the development of antimicrobial resistance.^5^ The prevalence of MGEs, including intracellular types (e.g., insertion sequences (IS) elements, transposons, and integrons) and intercellular types (e.g., conjugative and mobilizable plasmids, prophages, integrative conjugative elements (ICEs), integrative mobilizable elements (IMEs), underscores their crucial role in bacterial evolution and adaptation to changing environments.^6^^,^^7^ *Acinetobacter* species are well known opportunistic pathogens, inhabiting various environments such as hospital settings, human and animal wastewater and environmental waters. Infection caused by multidrug-resistant *Acinetobacter* has been a significant concern. Research focusing on the frequency of MGEs and their associated antibiotic resistance genes (ARGs) is essential to mitigate the risk of multidrug resistance development in the genus *Acinetobacter.*

While MGEs are present across all bacterial species, the frequency of different MGE types can vary significantly depending on the host bacterium’s ecology and environment. For instance, prophages, an intercellular MGE type, display variable abundance between *Staphylococcus aureus* isolates, being present in human isolates but absent in pig isolates.^8^ Similarly, plasmids, which are extrachromosomal MGEs, are frequently detected in livestock wastes.^9^ The abundance of MGE types is also influenced by the presence of defense systems that control HGT.^10^^,^^11^ Bacteria utilize sophisticated defense systems, such as CRISPR-Cas^12^^,^^13^ and restriction modification systems,^14^ which recognize specific DNA sequences to target phages and plasmids.

The prevalence of ARGs varies across different MGE types. Previous studies have shown that ARG prevalence is primarily elevated in intracellular MGEs types, such as transposable elements (TEs).^15^^,^^16^ Among intracellular MGE types, IS elements have been shown to have higher ARG prevalence compared to integrons.^7^ Among intercellular MGE types, higher ARG prevalence has been reported in plasmids compared to prophages.^17^^,^^18^ Plasmids can be categorized as conjugative, mobilizable, or nonmobilizable depending on the presence of conjugative genes such as relaxase and conjugative coupling proteins.^19^ Although plasmids are well-known ARG vectors, over 70% of plasmids lack ARGs.^20^ Based on these observations, we hypothesize that plasmids harbor a high frequency of ARGs due to the presence of IS elements that accumulate ARGs.

Nested MGE structures, or intracellular MGEs embedded within an intercellular MGE, mediate both intracellular and intercellular ARG transfer, forming sophisticated networks of gene transfer.^21^^,^^22^^,^^23^ One example of nested MGE structure is a plasmid harboring IS elements. Since IS elements are only capable of intracellular movement, a nested MGE structure is vital for their efficient intercellular transfer. For example, IS elements can mediate the intracellular transfer of ARGs from chromosomes to plasmids, while the plasmids that receive these ARGs can mediate the intercellular transfer of ARGs. Notably, IS elements demonstrate distinct distribution patterns between genomic compartments, with plasmids showing significantly higher IS density compared to chromosomal regions.^24^ However, the frequency of IS elements among different types of intercellular MGEs has yet to be fully characterized.

Genomic resistance island (GRI) is a variable region in the bacterial genome formed through intracellular and intercellular ARG transfer.^25^ These regions integrate MGEs, including integrons and transposons, along with associated ARGs. In *Acinetobacter baumannii*, GRIs have been extensively characterized, contributing to the development of multidrug resistance by harboring different ARGs.^25^^,^^26^^,^^27^^,^^28^^,^^29^^,^^30^^,^^31^^,^^32^^,^^33^ However, while studies have documented GRIs in *A. baumannii* from several parts of the world, knowledge gaps persist regarding the types of GRIs in non-*baumannii* species. Recent investigations have identified genomic islands (GIs) known as *A. baumannii* resistance islands (AbaR)-type GRIs in non-*baumannii* species, such as *A. nosocomialis* and *A. seifertii*, demonstrating interspecies transfer capabilities.^34^ Notably, a significant knowledge gap exists regarding the characteristics and prevalence of GRIs in non-*baumannii* species, particularly the pathogenic *Acinetobacter calcoaceticus—baumannii* (Acb) complex group.

The present study examines the mechanisms of ARG mobility in *Acinetobacter* species from various sources (i.e., patient, environmental, and pig waste samples), through comprehensive *in silico* analysis of genomic sequences, with particular emphasis on MGEs and their role in ARG acquisition and dissemination. The aim of this study is to characterize the hierarchical process of ARG transfer mediated by intracellular and intercellular MGEs. Specifically, we investigated whether the frequencies of MGE types in *Acinetobacter* species are influenced by source origins and their defense systems. Additionally, we examined the ARG prevalence among different types of MGEs and the co-localization patterns of ARGs within MGEs. Subsequently, we also analyzed the nested MGE structures and investigated which type of intercellular MGEs exhibits a high IS element frequency. Based on these results, we tested the hypothesis that ARGs are frequently present in plasmids because IS elements frequently harbor ARGs and are highly abundant in plasmids. Lastly, we discuss the intracellular and intercellular movement of ARGs and how it contributes to the formation of GIs in *Acinetobacter.*

## Results

### Prevalence of mobile genetic elements in *Acinetobacter* isolates

**Figure 1:**
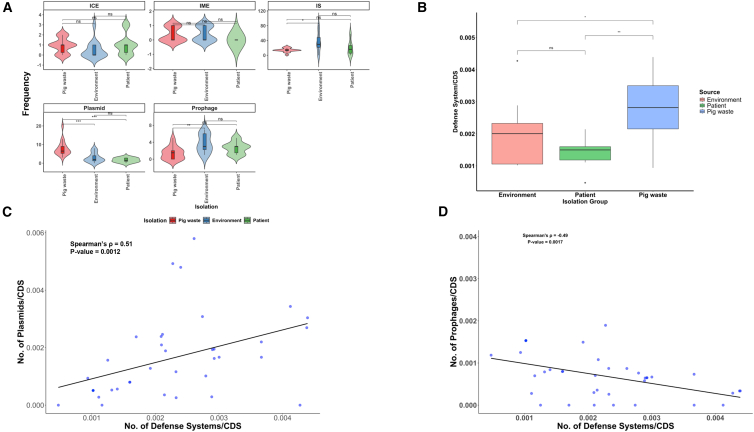
Frequency of mobile genetic elements (MGEs) and defense systems across different isolation sources (A) The frequency of integrative conjugative elements (ICEs) and integrative mobilizable elements (IMEs) in *Acinetobacter* chromosomes. Frequency of insertion sequence (IS) elements in the chromosome of *Acinetobacter* species (∗*p* < 0.05). Plasmid counts among patient-, pig waste- and environment-associated isolates (∗∗∗*p* < 0.001). Number of prophages integrated in the chromosome of *Acinetobacter* isolates (∗∗*p* < 0.01). Data are presented as median with interquartile range. (B) The frequency of defense systems in *Acinetobacter* species across different isolation sources (*p∗*<0.05, ∗∗*p* < 0.01). Kruskal-Wallis test was performed to compare the average number of MGEs and defense systems among *Acinetobacter* isolates. Data are presented as median with interquartile range. (C) Correlation plot of the number of plasmids and number of defense systems using Spearman’s rank correlation (R = 0.51, *p* = 0.0012). (D) Correlation plot of the number of prophages and number of defense systems using Spearman’s rank correlation (R = −0.49, *p* = 0.0017). Each dot represents a genome (*n* = 38).

Of the 38 *Acinetobacter* isolates examined, IS elements (*n* = 1045) were the most prevalent MGEs, followed by plasmids (*n* = 193) and prophages (*n* = 103) (Figure S1A). ICEs and IMEs were the least prevalent (*n* = 28 and *n* = 13, respectively). Significant differences in the frequencies of each MGE type were observed between environment- and pig waste-associated isolates, specifically for IS, prophage, and plasmid (Figure 1A). However, there was no significant difference in the frequency of ICE and IME carriage among *Acinetobacter* isolates. IS elements were significantly more abundant in environment-associated isolates than pig waste-associated isolates (*p* = 0.01, Kruskal-Wallis). Additionally, environment-associated isolates had more prophages than pig waste-associated isolates (*p* < 0.01, Kruskal-Wallis). In contrast, plasmids were significantly more prevalent in pig waste-associated isolates than in patient- and environment-associated isolates (*p* < 0.0001, Kruskal-Wallis). We also observed a significant difference in the mean frequencies of defense systems among *Acinetobacter* isolates from different sources. Pig waste-associated isolates had a higher frequency of defense systems than both environment- (*p* < 0.05, Kruskal-Wallis) and patient-associated isolates (*p* < 0.01, Kruskal-Wallis) (Figure 1B). Furthermore, we observed a significant positive correlation (*p* = 0.001, Spearman correlation) between the number of plasmids and the number of defense systems (Figure 1C). Interestingly, we also found a significant inverse correlation (*p =* 0.002, Spearman correlation) between the number of defense systems and the number of prophages in the *Acinetobacter* genomes (Figure 1D). Our analysis also revealed a distinct distribution pattern of defense systems between bacterial chromosomes and plasmids. Specifically, plasmids predominantly harbor BREX_I, SanaTA, and Retron_VI systems, while chromosomes encode diverse array of defense mechanisms including Gabija, Gao_Mza, multiple restriction modification (RM) types (I, II, IIG, III, and IV), and Wadjet_I (Figure S2).

### ARG distribution, co-occurrence, and enrichment in plasmids

The extent of ARG carriage in different MGE types was also investigated. Most ARGs were found to be associated with 42 plasmids and one integrative mobilizable element (IME), with no ARGs detected in prophages or ICE regions. A total of 193 plasmids were identified in the 38 *Acinetobacter* isolates. Most of the isolates’ plasmid repertoire consists of nonmobilizable plasmids (*n* = 105, 54.4%), mobilizable plasmids (*n* = 81, 42.0%), and conjugative plasmids (*n* = 7, 3.6%) (Figure S3A). Plasmid sizes exhibited remarkable diversity, ranging from 1.4 kb to 657 kb (Figure S3B). One hundred thirty-nine plasmids (72%) could not be assigned to a plasmid typing scheme using the rep gene (Figure S3C). Similarly, relaxase were not detected in 105 plasmids (54.0%) using the mob typing scheme (Figure S3D).

**Figure 2:**
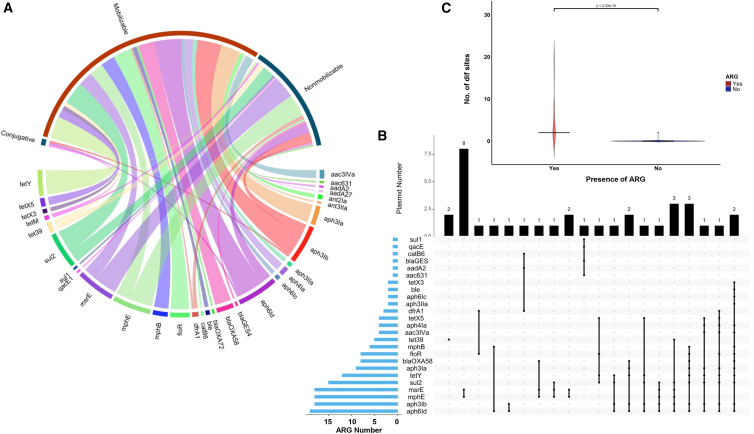
ARG distribution, co-occurrence, and enrichment in *Acinetobacter* plasmid (A) Chord diagram showing the association of ARGs with different plasmid types. (B) Upset plot showing the ARG number in blue bar plots, ARG co-localization patterns shown in interconnected dots and number of plasmids having the ARG co-localization pattern in black bar plots. (C) Comparison of the number of p*dif* sites between ARG-bearing plasmids and plasmids without ARGs (*p* < 2.22e−16). Wilcoxon test was performed to compare the average number of IS elements and number of p*dif* sites between ARG-bearing plasmids (red) and plasmids without ARGs (blue). Data are presented as median with interquartile range.

A total of 42 plasmids were detected harboring various ARGs (Figure 2A). The majority (40 out of 193 plasmids; 95.2%) were found in pig waste-associated isolates. Large size plasmids (>100 kb) tend to harbor more ARGs (2–16 ARGs) and were categorized as mobilizable or nonmobilizable. The proportion of plasmids harboring ARGs was 28.6% (2/7) among conjugative plasmids, 29.6% (24/81) among mobilizable plasmids, and 15.2% (16/105) among nonmobilizable plasmids (Table S2). We revealed different patterns of ARG co-localization in plasmids (Figure 2B). Macrolide resistance genes (*mph(E)* and *msr(E*)) and aminoglycoside resistance genes (*aph(3″)-Ib* and *aph(6)-Id*) tend to co-occur in 40.5% and 38.1% of ARG-carrying plasmids, respectively. While *sul2* and *aph(6)-Id* and *bla*_OXA-58_ and *aph(3″)-Ia* tend to co-occur in 33.3% and 16.67% of ARG-carrying plasmids, respectively. Further analysis revealed that 19 pairs of ARGs were co-localized on a single plasmid, showing significant associations (*p* = <0.05, using Fisher’s test) (Table S3). Up to 14 different ARGs were detected on a single plasmid. Nine prophage regions were also detected in eight plasmids. However, no ARGs were directly encoded within the prophage regions of these plasmids.

We assessed the presence of p*dif* sites or plasmid site-specific recombination sites in all ARG-carrying plasmids, as well as investigating whether these sites were associated with the acquisition of ARGs. As many as 20 p*dif* sites were detected in a single ARG-bearing plasmid. Multiple ARG types were flanked by these p*dif* sites, forming ARG p*dif* modules (Figures S4A–S4G). In total, seven different ARG p*dif* modules were identified: p*dif-*(*msr(E)-mph(E)*), p*dif*-(*tet(39)*), p*dif*-(*bla*_OXA-58_), p*dif*-(*aph(3″)-Ib – aph(6)-Id – tet(Y) – aph(3″)-Ia*), p*dif*-(*aph(3″)-Ia – tet(Y) – aph(6)-Id – aph(3″)-Ib – floR – mph(B) – sul2*), p*dif*-(*sul2 – aph(3″)-Ib – aph(6)-Id*), and p*dif*-(*aph(4)-Ia – aac(3)-IVa – aph(3″)-Ia – aph(3″)-Ib – aph(6)-Id – tet(Y) – tet(X5) – floR – dfrA1 – sul2*). The number of ARGs in p*dif* modules ranged from 1 to 10 per module. Plasmids with ARGs had more p*dif* sites than those without ARG (*p* = 2.2 × 10^−16^, Wilcoxon test) (Figure 2C). Furthermore, there was a significant association between the number of p*dif* sites and the number of ARGs in ARG-carrying plasmids (*p* = 2.3 × 10^−25^, using Fisher’s test).

### Nested MGE structures in *Acinetobacter*

**Figure 3:**
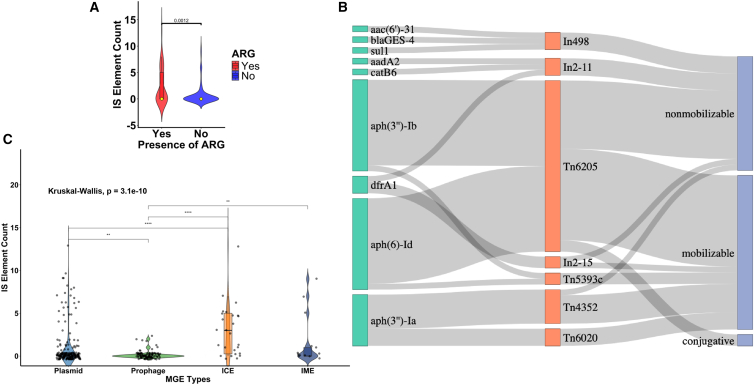
Nested MGE structures in *Acinetobacter* (A) Comparison of IS element carriage between ARG-bearing plasmids and plasmids without ARGs (*p* = 0.0012). Data are presented as median with interquartile range. (B) Sankey plot showing the association of antimicrobial resistance genes (green) in nested MGEs—transposable elements (orange) and different plasmid types (blue). (C) Frequency of IS elements among different intercellular MGE types (∗∗*p* < 0.01, ∗∗∗∗*p* < 0.0001). Kruskal-Wallis test was performed to compare the frequency of IS elements in A and C. Data are presented as median with interquartile range.

A total of 29 types of IS elements were detected among 193 plasmids (Figure S5A). The most prevalent IS elements were ISAba14 (*n* = 23) and IS17 (*n* = 23), followed by ISOur1 (*n* = 20), ISAba34 (*n* = 19), and IS1006 (*n* = 18). We examined whether ARG enrichment in plasmids is associated with the presence of IS elements. Plasmid-bearing ARGs possessed more IS elements compared to plasmids without ARGs (*p =* 0.0012, Wilcoxon test) (Figure 3A). The number of IS elements was positively correlated with the plasmid size (Figure S5B). The Sankey plot analysis showed that nine ARGs existed in TEs on plasmids (Figure 3B). The aminoglycoside resistance genes, *aph(6)-Id* and *aph(3″)-Ib*, both occurred on Tn6205, while *aph(3″)-Ia* was found on Tn4352. Furthermore, ARGs such as *dfrA1*, *bla*_GES-4_, *aac(6′)-31*, *aadA2*, and *catB6* were identified within gene cassettes associated with integrons.

Among the four types of intercellular MGEs, IS elements were predominantly found in conjugative elements such as plasmids and ICEs. The frequency of IS carriage was significantly different between plasmids and prophages (*p* < 0.0001, Kruskal-Wallis), as well as between ICEs and prophages (*p* < 0.001, Kruskal-Wallis) (Figure 3C). The frequency of IS elements also varies among plasmid types, with mobilizable and non-mobilizable plasmids possessing more IS elements (Figure S5C). Furthermore, no significant difference was observed in the frequency of IS element carriage among plasmids from different isolation sources (patient, pig waste, environment) (*p* = 0.6, Kruskal-Wallis) (Figure S5D). However, the types of IS elements varied among plasmids isolated from different isolation sources, with some types being exclusively found in plasmids from a specific isolation source (Figure S6).

Duplication of ARGs on the same plasmid was also observed. Some of the ARGs that showed duplication were aminoglycoside resistance genes, such as *aph(3″)-Ib*, *aph(6)-Id*, *ant(2″)-Ia*, and macrolide resistance genes *msr(E)*, and *mph(E)* (Table S4). Additionally, we revealed that several plasmids from different bacterial hosts possessed similar set of ARGs (Figure S7A) or possessed similar plasmids with the same set of ARGs and other functional genes (Figure S7B). We demonstrated the potential movement of macrolide resistance genes (*msr(E)* and *mph(E*)) and aminoglycoside resistance genes (*aph(3″)-Ib* and *aph(6)-Id*) between different plasmids in different hosts. A similar set of ARGs and functional genes was also observed between co-residing plasmids. Specifically, this was observed for the *aph(3″)-Ib* and *aph(6)-Id*, *sul2*, and *msr(E)* and *mph(E)* genes (Figures S8A–S8C).

Nine prophage regions, ranging in size from 44 to 183 kilobases, were identified across eight plasmids. Individual plasmids harbor from one or two prophages. Notably, these prophages were not associated with *Acinetobacter* as their host bacterium; rather, they represented various phage types, including Escher_RCS47, Stx2_c_1717, Escher_PA28, Entero_VT2phl, Stx2_II, Staphy_Spbeta, Faecal_FP_Taranis, and Salmon_epsilon15 (Table S5). Out of the 8 prophages detected in plasmids, 6 (75%) were found to be intact or complete. Furthermore, we observed that prophage regions in plasmids exhibited a significantly higher frequency of IS elements than those located within the chromosomes (*p =* 7.5e−13, Wilcoxon test) (Figure S9A). We also found that intact prophages had a significantly higher frequency of IS elements compared to incomplete prophages (*p =* 0.03, Kruskal-Wallis) (Figure S9B).

### Genomic islands in non-*baumannii* species

**Figure 4:**
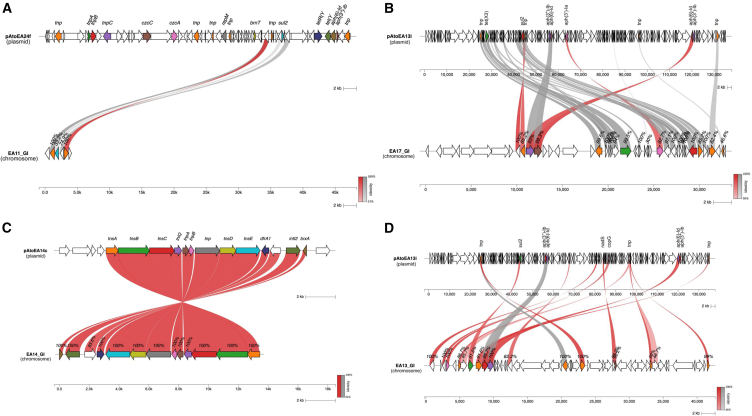
Genomic resistance islands (GRIs) in non-*baumannii* isolates (A) ARG synteny analysis showing homologous region of *sul2* gene between plasmid pAtoEA24f (*A. towneri*) and chromosome of *A. indicus* EA-11. (B) ARG movement from a co-residing plasmid pAtoEA13i (*A. towneri*) into the chromosome (*A. towneri* EA-13). (C) ARG synteny analysis showing homologous region of *dfrA1* gene in an integron of plasmid pAtoEA23i *A.* (*towneri*) and a plasmid pAtoEA14c from a different bacterial host (*A. towneri*). The same region was found integrated into the chromosome of *A. towneri* EA-14. (D) ARG synteny analysis of ARGs from plasmid pAtoEA13i (*A. towneri*) and genomic island in *A. haemolyticus* EA-17. Red lines indicate high sequence similarity matches while gray lines indicate reverse or inverted synteny.

We detected four putative GIs in non-*baumannii* pig waste-associated isolates possibly mediated by intracellular ARG transfer from plasmids to chromosomes. The ARG synteny analysis revealed that mobilizable ARG regions in plasmids of the pig waste-associated isolates showed high sequence similarity with the ARG regions in the chromosome of the host bacteria. Furthermore, TEs in plasmids showed homologous regions within the chromosome. The first GRI type was detected in a plasmid of *A. towneri* (EA24) flanked by IS1006 and containing a sulfonamide resistance gene, as well as cobalt, zinc, and cadmium resistance gene *czcA* gene (Figure 4A). The second GRI type, observed in *A. towneri* (EA13), was marked by an IS3 family-bound GRI containing resistance genes (*aph(3″)-Ib*, *aph(6)-Id*, and *sul2*), metal resistance genes (*copA*, *copB*, *merR*, *zitB*, *czcABD*), and a toxin/anti-toxin system (Figure 4B). The third GRI type included an integron class 2, associated with the Tn7 transposon family, and was identified in *A. towneri* (EA14), harboring a gene cassette bearing a trimethoprim resistance gene (*dfrA1*) (Figure 4C). The fourth GRI type, observed in *A. haemolyticus* (EA17), featured an ISAba1-bound GRI that contained aminoglycoside resistance genes (*aph(3″)-Ia*, *aph(3″)-Ib*, and *aph(6)-Id*); a tetracycline resistance gene (*tet(X3)*); and sulfonamide resistance gene (*sul2*) (Figure 4D).

## Discussion

MGEs are abundant, and their distribution varies in the bacterial genome. A notable differentiation in the distribution of MGEs based on the isolation source was observed in our study. Plasmids were abundant in pig waste-associated isolates, whereas they were rare in patient- or environment-associated isolates. These findings are consistent with previous studies, which have reported the widespread plasmids and demonstrated a strong positive correlation between the numbers of plasmids and ARGs per cell.^9^^,^^35^ Specifically, integrons and conjugative plasmids, which are widely distributed in livestock waste, serve as key vectors for ARG dissemination via HGT.^36^^,^^37^ The extensive use of antibiotics in pig farming imposes significant selective pressure, favoring bacterial populations that harbor ARG-bearing plasmids.^38^

In contrast, prophages were observed at a higher frequency in environment-associated isolates compared to those from patients or pig waste. This trend corresponds with prior studies that identified a high prevalence of prophages in environments such as wastewater treatment plants (WWTPs), which harbor diverse bacterial populations of human origin.^39^ A large-scale analysis of prophages from 13,713 complete prokaryotic genomes also identified genera such as *Acinetobacter*, *Enterobacter*, and *Pseudomonas* as major prophage reservoirs.^40^ Furthermore, it has been reported that prophages were frequently detected in human-derived *Staphylococcus aureus* isolates compared to those from pig origin.^8^ While this study revealed a significant difference in terms of MGE frequency among *Acinetobacter* isolates from different sources, the limited sample size precludes definitive conclusions about the general distribution of MGEs across the *Acinetobacter* population.

To further investigate the factors influencing the frequency of MGEs, we examined the presence of bacterial defense systems within each *Acinetobacter* genome. Notably, a negative correlation was observed between the number of defense systems and the number of prophages, highlighting a possible role of defense systems in restricting prophage acquisition. This observation is consistent with findings from Kohgay et al. who reported fewer phage-related genes in bacterial genomes with a higher abundance of defense systems.^41^ Similarly, Costa et al. demonstrated that an increase in defense systems enhances bacterial resistance to phage infections, further supporting their involvement in limiting prophage acquisition.^42^

Interestingly, we observed a compartmentalized organization of bacterial defense systems between the bacterial chromosomes and plasmids. Isolates derived from pig waste exhibited the highest frequency of defense systems and plasmids. Additionally, we observed a significant positive correlation between the numbers of defense systems and plasmids. Notably, the plasmid-specific enrichment of BREX_I, SanaTA, and Retron_VI systems were observed. Plasmids, while known as principal vectors of ARG dissemination, also carry diverse defense systems, which protect host bacteria from phage infections. Recent studies (emphasized the strong association between plasmids and bacterial defense systems, with plasmids playing a pivotal role in the accumulation and dissemination of these systems across bacterial genomes.^43^^,^^44^

Based on these observations, we propose a hypothetical model wherein isolates originating from pig waste environments acquire plasmids carrying multiple ARGs under the selective pressure of high antibiotic use. These plasmids may additionally harbor defense systems that protect the host bacteria against phages. Future experimental studies are needed to validate this hypothesis and further elucidate the interplay between plasmids, defense systems, and prophage dynamics in different environmental contexts.

Our analysis also revealed that ARGs in *Acinetobacter* isolates were predominantly associated with plasmids. Notably, the ARG carriage accounted for only 21% of the total plasmids identified across all isolates, consistent with former findings indicating that more than 70% of plasmids lack known resistance genes.^20^ Interestingly, most ARGs in this study were found in mobilizable and non-mobilizable plasmids, in contrast to other studies showing an enrichment of ARGs in conjugative plasmids.^45^ Distinct patterns of ARG co-localization were observed in plasmids. For instance, beta-lactamase gene *bla*_OXA-58_ was co-localized with aminoglycoside resistance gene (*aph*(*3*″)-*Ia*), while *sul2* was co-localized with *aph*(*6*)-Id. Such co-localization suggests mechanisms of co-resistance or the encoding of multidrug resistance, potentially promoting the survival and emergence of multidrug-resistant *Acinetobacter* strains. The conservation of such ARG clusters indicates that they may be transferred or inherited as a single unit.

Furthermore, ARG enrichment was identified within p*dif* modules in plasmids carrying ARGs. The *dif* site comprised a 28 bp DNA sequence featuring two 11 bp Xer protein binding motifs arranged in an inverted repeat configuration, with a 6 bp central region.^46^ In plasmids, this *dif* site is referred to as the p*dif* module, often occurring in multiple copies throughout the plasmid DNA. Previous studies have reported p*dif* module-associated ARG enrichment in *Acinetobacter* plasmids.^47^^,^^48^ However, this study, to the best of our knowledge, is the first to document up to 10 different ARGs localized within a single p*dif* module.

While ARGs are predominantly associated with TEs and plasmids, their occurrence within prophage regions showed considerable variation across bacterial species. Comprehensive genomic studies of clinical isolates generally report a lack of ARGs in the prophage regions of *Pseudomonas aeruginosa*,^49^ *Escherichia coli*,^50^ and *Staphylococcus aureus*.^51^ However, contrary findings have demonstrated ARG presence within prophage regions of *A. baumannii* isolates.^52^ In contrast to the findings of Loh et al.^52^ and consistent with earlier studies on other bacterial species,^49^^,^^50^^,^^51^ our analysis detected no ARGs in prophage regions of *Acinetobacter* genomes. This absence may be attributed to the size constraints of phages, which limit their ability to carry accessory genes like ARGs.^53^ Additionally, the discrepancy with previous studies may stem from advancements in prophage detection tools. Our study utilized the latest iteration of PHASTEST for prophage prediction, which integrates the Prodigal algorithm,^54^ characterized by reduced false-positive and false-negative rates for open reading frame (ORF) identification. Previous studies employed an earlier version of PHASTER, potentially leading to the detection of ARG-containing prophages that are not confirmed with the updated toolset. This difference highlights the need for methodological standardization in prophage analysis. Nonetheless, the limited presence of ARGs in prophage regions supports the notion that plasmids are the primary vehicles ARG transfer in *Acinetobacter*.

We then analyzed the potential role of the nested structure of MGEs in the intracellular and intercellular movement of ARGs *in silico*. We observed that ARGs in plasmids were primarily associated with intracellular MGEs, consistent with previous findings that ARGs are frequently linked to TEs and integrons.^16^ Although IS-harboring plasmids carrying ARGs accounted for only 8% of the analyzed plasmids, the presence of IS elements highlights their role in facilitating gene shuffling and replication both within and between plasmids.

IS elements can also transfer from plasmids to chromosomes, enabling bacteria to maintain HGT capabilities while reducing the metabolic burden with plasmid maintenance.^23^ IS elements linked to ARGs are key indicators of resistance transfer risk.^4^^,^^55^ Interestingly, we observed ARG-bearing plasmids had a higher frequency of IS elements compared to plasmids without ARGs. This aligns with previous studies showing that higher density of IS elements in plasmids is associated with ARG carriage.^5^^,^^56^ These findings may indicate that nested MGEs facilitate hierarchical ARG transfer, potentially promoting the movement of ARGs between plasmids, chromosomes, and even across cells, enhancing overall ARG dissemination.

We also examined the presence of prophages within plasmids, often referred to as “phage-plasmids,” which have been identified in notable pathogens such as *Escherichia*, *Acinetobacter*, *Klebsiella*, and *Salmonella*.^57^^,^^58^ For instance, in *E. coli*, the plasmid p1108-IncY exhibits high nucleotide sequence identity with phage P1, suggesting lysogenization via genetic integration.^59^ Similarly, prophage regions have been identified in *A. baumannii* plasmids.^60^ In our study, we detected phage-plasmids in non-*baumannii Acinetobacter* species, such as *A. towneri* and *A. guillouiae.*

Subsequently, our synteny analysis revealed a potential intracellular transfer of multiple ARGs from plasmids to chromosomes playing a significant role in the formation of GIs. These are variable chromosomal regions that, when enriched with resistance genes, are referred to as GRIs. This transfer is primarily mediated by TEs, especially when ARGs are co-localized with transposons or integrons. These findings suggest that ARG movement within bacterial cells (e.g., from plasmids to chromosomes) is a key driver in the formation of new GRIs. While these co-localizations suggest potential HGT, they represent indirect indicators rather than definitive proof of active gene movement. These findings should be interpreted cautiously as markers of shared mobile genetic structures rather than confirmed transfer events. Direct experimental verification through conjugation or transformation assays would be necessary to definitively establish the mobility of these genetic elements.

GRIs have been extensively studied in *Acinetobacter*, especially in clinical and pathogenic isolates. In *A*. *baumannii*, numerous GRIs have been identified and characterized.^27^^,^^28^^,^^30^^,^^31^^,^^61^^,^^62^^,^^63^^,^^64^ However, studies on GRIs in non-*baumannii* species are limited and have mainly focused on clinical isolates from the *Acinetobacter calcoaceticus-baumannii* (Acb) complex, including *A. nosocomialis* and *A. seifertii*.^34^

In this study, we identified GRIs in non-*baumannii* strains isolated from pig farm wastewater and discovered GRI structures that distinctly differ from those previously reported in clinical isolates.^30^^,^^31^^,^^62^ Remarkably, this represents the first report of GRIs in non-*baumannii* species outside the Acb complex. The discovery of these GRI structures in non-*baumannii* isolates highlights their adaptation to distinct selective pressures encountered in non-clinical environments, such as those characteristics of pig waste. Plasmids isolated from pig waste-associated isolates exhibited a notable enrichment of distinct ARG types not commonly observed in patient- and environment-associated isolates. Transposition of these ARGs from plasmid to the chromosome perhaps gives rise to the formation of putative GRIs.

### Limitations of the study

This work has several limitations that should be acknowledged when interpreting the findings. The relatively small number of *Acinetobacter* isolates (i.e., number of clinical isolates) reduces statistical power, hindering the detection of rare MGEs and low-frequency ARG-MGE associations, and it limits the generalizability of the results across different ecological niches, geographic regions, or clinical contexts. Furthermore, the reported relationships among MGEs, ARGs, and defense systems are correlative and do not establish causality. Experimental validation is required to determine whether the co-occurrence reflects direct mechanistic interactions or is driven by shared selective pressures or population structure. Overall, these limitations underscore the need for larger, more diverse datasets and complementary experimental approaches to strengthen confidence in the inferred ARG-MGE dynamics.

## Resource availability

### Lead contact

Request for additional information or resources should be addressed to the lead contact, Professor Kozo Watanabe (watanabe.kozo.mj@ehime-u.ac.jp), who will handle all such inquiries.

### Materials availability

Materials will be made available upon request.

### Data and code availability

• Data: The study did not generate new set of sequences.
• Code: This paper does not report any original code. All details related to supplementary software used for the analysis are listed in the key resources table.
• All other items: The study sought approval from the Ehime University Hospital Clinical Research Ethics Review Committee with approval number 2511001. Any further details necessary to reproduce the data presented in this study can be obtained from the corresponding lead contact upon request.

## Acknowledgments

This research was performed by the Environment Research and Technology Development Fund (SII-12-1 (3)) of the 10.13039/100014423Environmental Restoration and Conservation Agency provided by 10.13039/501100006120Ministry of the Environment of Japan. This research was supported by the 10.13039/501100001691Japan Society for the Promotion of Science (10.13039/501100001691JSPS) Grant-in-Aid for Scientific Research (A) (20H00633).

## Author contributions

Conceptualization, K.B., S.S., and K.W.; methodology, K.B., N.K., H.T., and S.S., investigation, K.B., formal analysis, K.B., N.K., and K.W.; writing – original draft, K.B.; writing-reviewing and editing, K.B., N.K., H.T., S.S., and K.W.; project administration, K.B. and K.W. visualization; K.B.; funding acquisition, K.W.; supervision, S.S. and K.W.

## Declaration of interests

The authors declare no competing interests.

## STAR★Methods

### Key resources table

**Table:** 

REAGENT or RESOURCE	SOURCE	IDENTIFIER
**Bacterial and virus strains**		

*Acinetobacter baumannii*	Bongulto et al.^65^	BioProject Accession: PRJNA1184881
*Acinetobacter pittii*	Bongulto et al.^65^	BioProject Accession: PRJNA1184881
*Acinetobacter junii*	Bongulto et al.^65^	BioProject Accession: PRJNA1184881
*Acinetobacter guillouiae*	Bongulto et al.^65^	BioProject Accession: PRJNA1184881
*Acinetobacter bereziniae*	Bongulto et al.^65^	BioProject Accession: PRJNA1184881
*Acinetobacter ursingii*	Bongulto et al.^65^	BioProject Accession: PRJNA1184881
*Acinetobacter calcoaceticus*	Bongulto et al.^65^	BioProject Accession: PRJNA1184881
*Acinetobacter indicus*	Bongulto et al.^65^	BioProject Accession: PRJNA1184881
*Acinetobacter amyesii*	Bongulto et al.^65^	BioProject Accession: PRJNA1184881
*Acinetobacter johnsonii*	Bongulto et al.^65^	BioProject Accession: PRJNA1184881
*Acinetobacter towneri*	Bongulto et al.^65^	BioProject Accession: PRJNA1184881
*Acinetobacter haemolyticus*	Bongulto et al.^65^	BioProject Accession: PRJNA1184881

**Chemicals, peptides, and recombinant proteins**		

Blunt/TA Ligase Master Mix	New England BioLabs	M03367L
NEBNext® Companion Module for Oxford Nanopore Technologies Ligation Sequencing	New England BioLabs	E7180S
NEBNext® Quick Ligation Reaction Buffer	New England BioLabs	B6058S
NEBNext® FFPE Repair Mix	New England BioLabs	M6630S
NEBNext® Ultra II End repair/dA-tailing Module	New England BioLabs	E7546S
NEBNext® Quick Ligation Module	New England BioLabs	E6056S
Oxford Nanopore Technologies Ligation Sequencing Kit	Oxford Nanopore Technologies	SQK-LSK109
Flow Cell R9	Oxford Nanopore Technologies	FLO-MIN106D
Flow Cell Wash Kit	Oxford Nanopore Technologies	EXP-WSH004
Native Barcoding Expansion 1-12 (PCR free)	Oxford Nanopore Technologies	EXP-NBD104
Ampure XP Beads	Beckman Coulter Inc.	A63880

**Critical commercial assays**		

Illumina NovaSeq 6000 Whole Genome Sequencing	Eurofins Genomics	https://eurofinsgenomics.jp/jp/home/

**Deposited data**		

Whole genome sequences	Bongulto et al.^65^	BioProject Accession: PRJNA1184881

**Software and algorithms**		

MOB-suite (v3.1.8)	Robertson et al.^66^	https://github.com/phac-nml/mob-suite
Blastn		
*Acinetobacter* Plasmid Typing (APT) scheme (v3.0)	Lam et al.^67^	https://github.com/MehradHamidian/AcinetobacterPlasmidTyping
PHASTEST	Wishart et al.^68^	https://phastest.ca
MobileElementFinder	Johansson et al.^69^	https://cge.food.dtu.dk/services/MobileElementFinder/
BacAnt	Hua et al.^70^	bacant.net/BacAnt/
Integron Finder	Néron et al.^71^	https://github.com/gem-pasteur/Integron_Finder
ICEberg (v2.0)	Liu et al.^72^	http://db-mml.sjtu.edu.cn/ICEberg/
IslandViewer 4	Bertelli et al.^73^	https://www.pathogenomics.sfu.ca/islandviewer
pdifFinder	Shao et al.^74^	pdif.dmicrobe.cn/pdif/home/
Abricate	Seeman, T.^75^	https://github.com/tseeman/abricate
CARD (v3.2.4)	Alcock et al.^76^	https://github.com/arpcard/rgi
ResFinder (v4.6.0)	Florensa et al.^77^	https://github.com/genomicepidemiology/resfinder
DefenseFinder	Tesson et al.^78^	https://github.com/mdmparis/defense-finder
Bakta (v5.0)	Schwengers et al.^79^	https://github.com/oschwengers/bakta
Geneviewer (v0.1.6)	Van der Velden, N.^80^	https://nvelden.github.io/geneviewer/
R v4.2.0	R core Team, 2021	https://posit.co/download/rstudio-desktop/
RStudio (v2023.06.0+421)	RStudio Team, 2020	https://posit.co/download/rstudio-desktop/

### Experimental model and study participant details

#### Microbe strains

The study involves 38 *Acinetobacter* strains from different isolation sources. Nine strains including *A. baumannii, A. junii, A. pittii, A. guillouiae, A. bereziniae,* and *A. ursingii* were obtained from the Ehime University Hospital. All clinical isolates were prepared in glycerol stocks and were anonymized. Eighteen strains such as *A. indicus, A. haemolyticus, A. johnsonii, A. amyesii,* and *A. towneri* were isolated from pig wastewater sample. One strain was isolated from the river water (*A. pittii*) and coastal area (*A. johnsonii*). While 9 strains including *A. baumannii, A. junii, A. calcoaceticus,* and *A. pittii* were isolated from municipal wastewater sample.

#### Bioethical clearance

The study sought approval from the Ehime University Hospital Clinical Research Ethics Review Committee with approval number 2511001. The clinical isolates from glycerol stock cultures used in this study were anonymized.

### Method details

#### Data collection

The datasets, namely plasmid sequences and chromosome sequences of 38 *Acinetobacter* species from the different co-selection patterns of ARGs and virulence genes,^65^ were retrieved from the NCBI database under the BioProject accession number PRJNA1184881. We note that the previous study did not probe into the composition and association of MGEs and ARGs. Our set of 38 *Acinetobacter* isolates originated from patients (*n*=6), pig wastewater (*n*=18), municipal wastewater (*n*=9), natural surface water (*n*=2), and hospital environment (*n*=3) (Table S1). We categorized the 38 *Acinetobacter* strains into patient-associated (*n*=6), pig waste-associated (*n*=18), and environment-associated (*n*=14) groups. The patient-associated isolates include strains isolated from patients’ sputum, stool, and saliva. Strains isolated from hospital environment, municipal wastewater treatment plants, river, and coastal area were classified into the environment-associated isolates. Lastly, strains isolated from the pig farm wastewater were considered as pig waste-associated isolates since the influent sample primarily originated from pigs’ waste.

Two complementary sequencing approaches were employed in the previous study.^65^ First, long-read sequencing was performed using Oxford Nanopore Technology, where genomic DNA (∼1μg) was subjected to library preparation following the Oxford Nanopore Technologies’ protocol for environmental bacteria. The DNA library was prepared using the Ligation Sequencing Kit protocol (SQK-LSK109) and the long-read sequences were generated using the MinION FLO-MIN106D flow cell and a MinION MK1B sequencing device (Oxford Nanopore Technology). Finally, MinKNOW v23.07.12 software was used for data acquisition. To complement the long-read sequencing data, short-read sequencing was conducted using a NovaSeq 6000 S4 Reagent Kit (2 x 150 bp paired-end read setting) and sequencing was performed on an Illumina NovaSeq 6000 sequencer at Eurofins Genomic Ltd.

The hybrid assembled genome of *Acinetobacter* isolates were used to extract MGE sequences. The plasmid dataset (*n*=193) only includes circularized DNA and complete sequences. Other intercellular MGEs (e.g. prophages, integrative conjugative elements, integrative mobilizable elements) were extracted from the chromosome sequences. Further, chromosome and plasmid sequences were used to identify intracellular MGEs, such as IS elements, transposons, and integrons.

### Quantification and statistical analysis

#### Bioinformatics analysis

Plasmid identification was conducted by predicting the mob and rep genes. Plasmid types were predicted using the parameter mob_typer in MOB-suite v3.1.8^66^ which searched for the presence of the oriT, relaxase, and mate-pair formation in the plasmid sequence. Plasmids possessing either oriT or relaxase but missing the mate-pair formation were considered as mobilizable, while plasmids without an oriT and relaxase were considered nonmobilizable. Plasmid rep typing was conducted using blastn^81^ with the parameter -perc_identity 95 against the *Acinetobacter* Plasmid Typing (APT) scheme v3.0.^67^ Prophage regions in both chromosomes and plasmids were predicted using the default parameter of PHASTEST.^68^ MobileElementFinder^69^ was used to identify IS elements. Transposons were detected using BacAnt^70^ with the minimum identity threshold of 90% and 60% coverage. Integrons were detected using the parameter –local-max in Integron Finder v.2.0^71^ to allow for a more sensitive search and minimize the false positive rate. Integrative conjugative elements and integrative mobilizable elements were predicted using the default parameter of ICEberg 2.0.^72^ Genomic islands and p*dif* modules were predicted using the default parameter of IslandViewer 4^73^ and pdifFinder,^74^ respectively. To predict ARGs in MGE sequences, Abricate^75^ was used with the CARD v3.2.4^76^ and ResFinder v.4.6.0^77^ databases with a minimum identity threshold and minimum coverage threshold of 90%. Lastly, defense genes were predicted using the DefenseFinder tool.^78^

#### Gene synteny analysis

Each MGE sequence was annotated using Bakta^79^ and the full database version (v.5.0) was used to obtain the best annotation results. The annotated .gbk files were used for the gene synteny analysis using the R package geneviewer v0.1.6.^80^ The sequences of ARG-bearing plasmids and genomic islands in chromosome were used for the ARG synteny analysis.

#### Visualization and statistical analysis

The frequency of IS elements, plasmids, prophage regions, integrative conjugative elements (ICEs), and integrative mobilizable elements (IMEs), as well as defense systems among patient-, pig waste-, and environment-associated *Acinetobacter* isolates were determined using Kruskal-Wallis test with post hoc Wilcoxon rank-sum test. Statistical significance was considered at *p* <0.05. Additionally, the correlation between the number of plasmids and number of defense systems, and between number of prophages and number of defense systems were examined. The number of defense systems, plasmids, and prophages were normalized using the total number of protein-coding sequences (CDS). The correlations were evaluated using the cor function in R v.4.2.0. with statistical significance set at *p* < 0.05. The significant ARG co-localization on a single plasmid was tested using Fisher’s exact test with fisher.test function in R. The test was performed for all plasmid-bearing ARGs. The upset plot for the co-localization patterns of ARGs were generated in R v.4.2.0. Kruskal-Wallis test was conducted in comparing the frequency of insertion sequence (IS) elements across different intercellular MGEs such as plasmids, prophages, ICEs, and integrative mobilizable elements (IMEs) using the function kruskal.test in R v.4.2.0. Likewise, Wilcoxon rank test was also performed to determine the significant difference in terms of IS element carriage between plasmid-bearing ARGs and plasmid without ARGs. Significance levels are indicated as: ∗∗∗∗*p* <0.0001, ∗∗∗*p* <0.001, ∗∗*p* <0.01, and ∗*p* <0.05. Further, the relationship between p*dif* modules and ARG carriage in plasmid-bearing ARGs and plasmids without ARGs were investigated. Wilcoxon test was also performed with statistical significance set at *p* <0.05. Sankey plot was built using the networkD3 package in R to show the association of ARGs, intracellular MGEs, and intercellular MGEs.

## Supplemental information

Document S1. Figures S1–S9 and Tables S2, S3, and S5

Table S1. Species identification, isolation source, and antimicrobial resistance genes composition of the 38 Acinetobacter species

Table S4. Antimicrobial resistance genes (ARGs) in plasmids ARG duplication in plasmids were colored blue.
