# Supplementary material for: Nested mobile genetic elements mediating antimicrobial resistance genes mobility within and between Acinetobacter isolates
Source: iScience. 2026 Apr 8;29(5):115648. doi: 10.1016/j.isci.2026.115648 (PMC13125923; doi:10.1016/j.isci.2026.115648)
Supplement: Document S1. Figures S1–S9 and Tables S2, S3, and S5 [file mmc1.pdf]

iScience, Volume *29*

Supplemental information

Nested mobile genetic elements mediating antimicrobial resistance genes mobility within and between *Acinetobacter* isolates

Kenneth Bongulto, Ngure Kagia, Hisamichi Tauchi, Satoru Suzuki, and Kozo Watanabe

Supplementary Fig. 1.

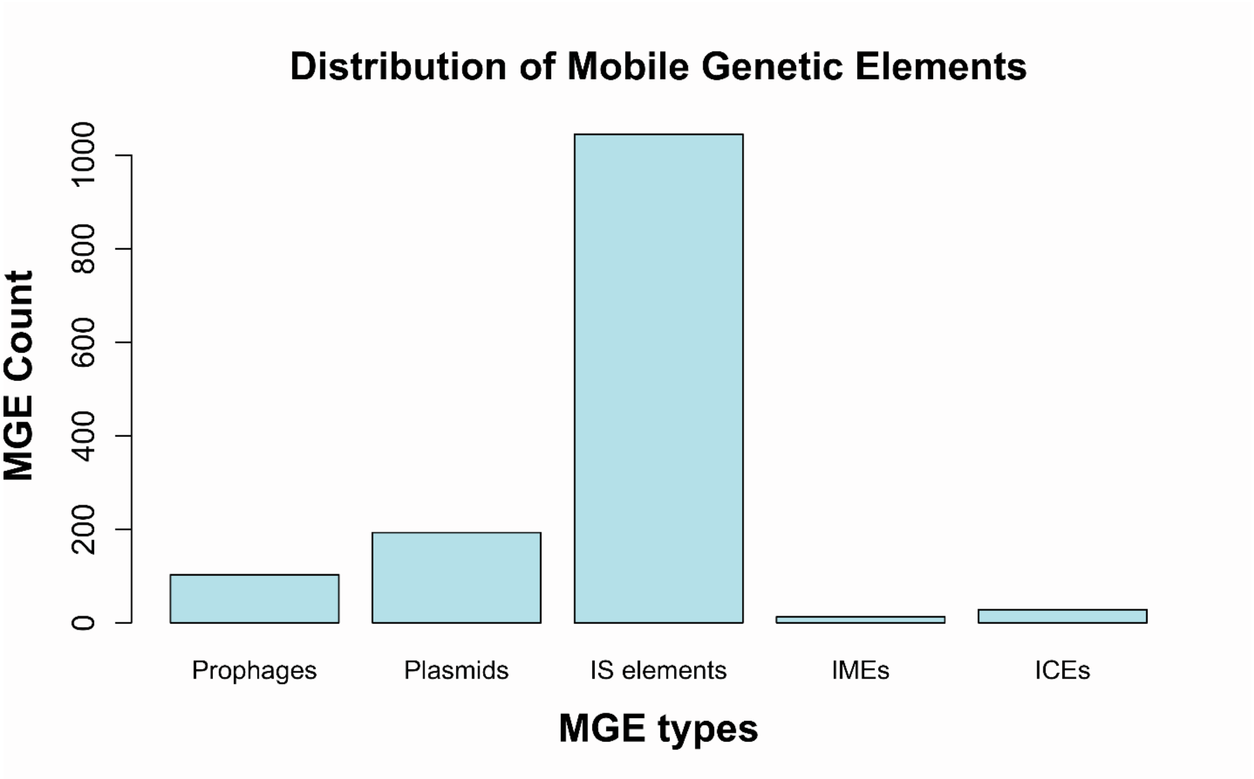

Supplementary Figure 1. Frequency of mobile genetic element (MGE) types. IS elements (n=1045), plasmids (n=193), prophages (n=103), integrative conjugative elements (ICEs) (n=28), and integrative mobilizable elements (IMEs) (n=13).

Supplementary Fig. 2.

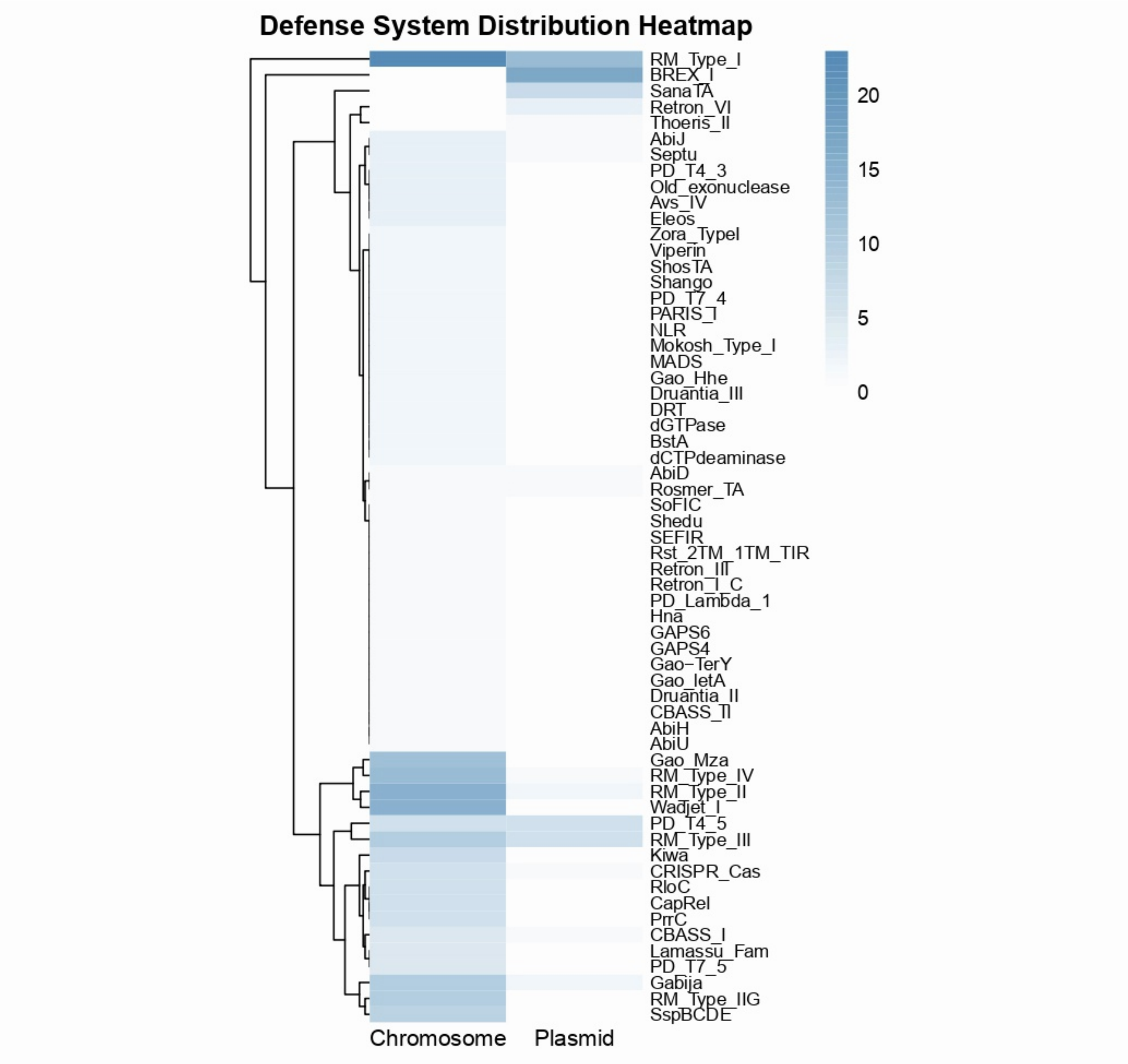

Supplementary Figure 2. Defense systems in *Acinetobacter* species. Distribution of bacterial defense systems in the chromosomes and plasmids.

Supplementary Fig. 3.

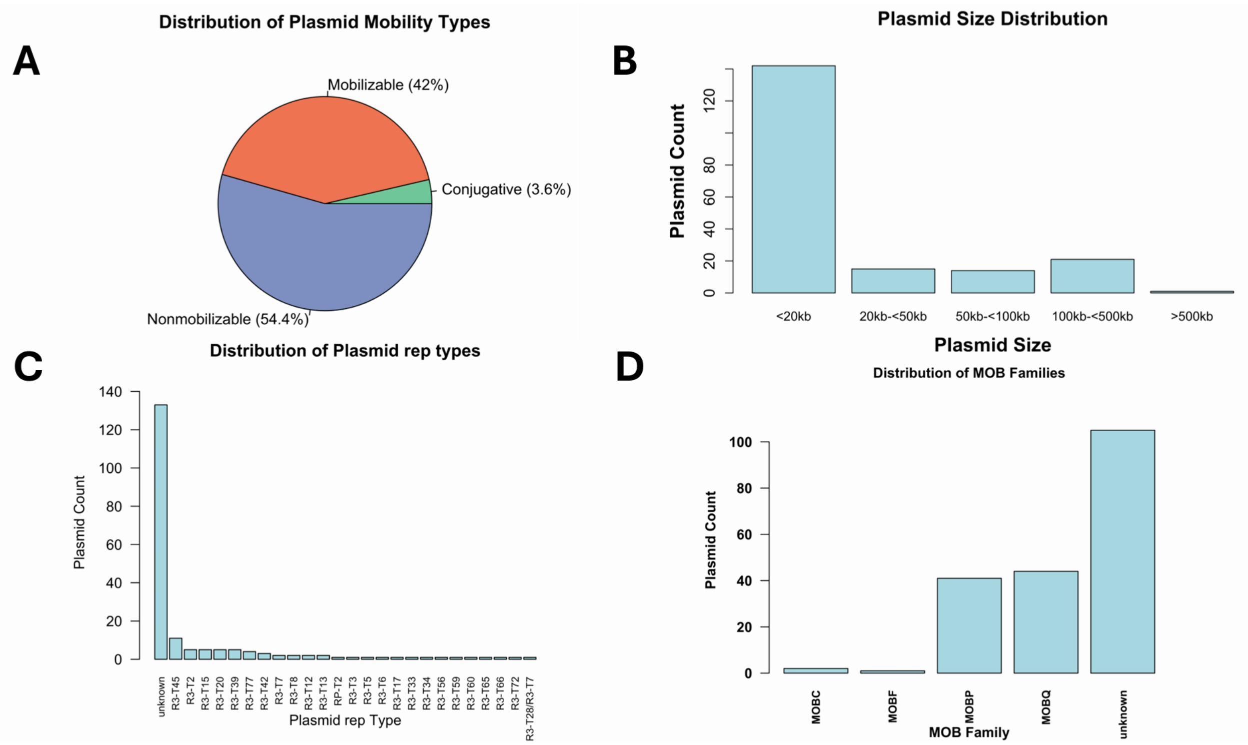

Supplementary Figure 3. Plasmid characterization ($_{plasmid}$ *n*= 193). A. Distribution of plasmids based on plasmid mobility. **B.** Distribution of plasmids based on plasmid size. **C.** Distribution of plasmids based on *rep* gene typing. **D.** Distribution of plasmid MOB family based on *mob* module.

Supplementary Figure 4.

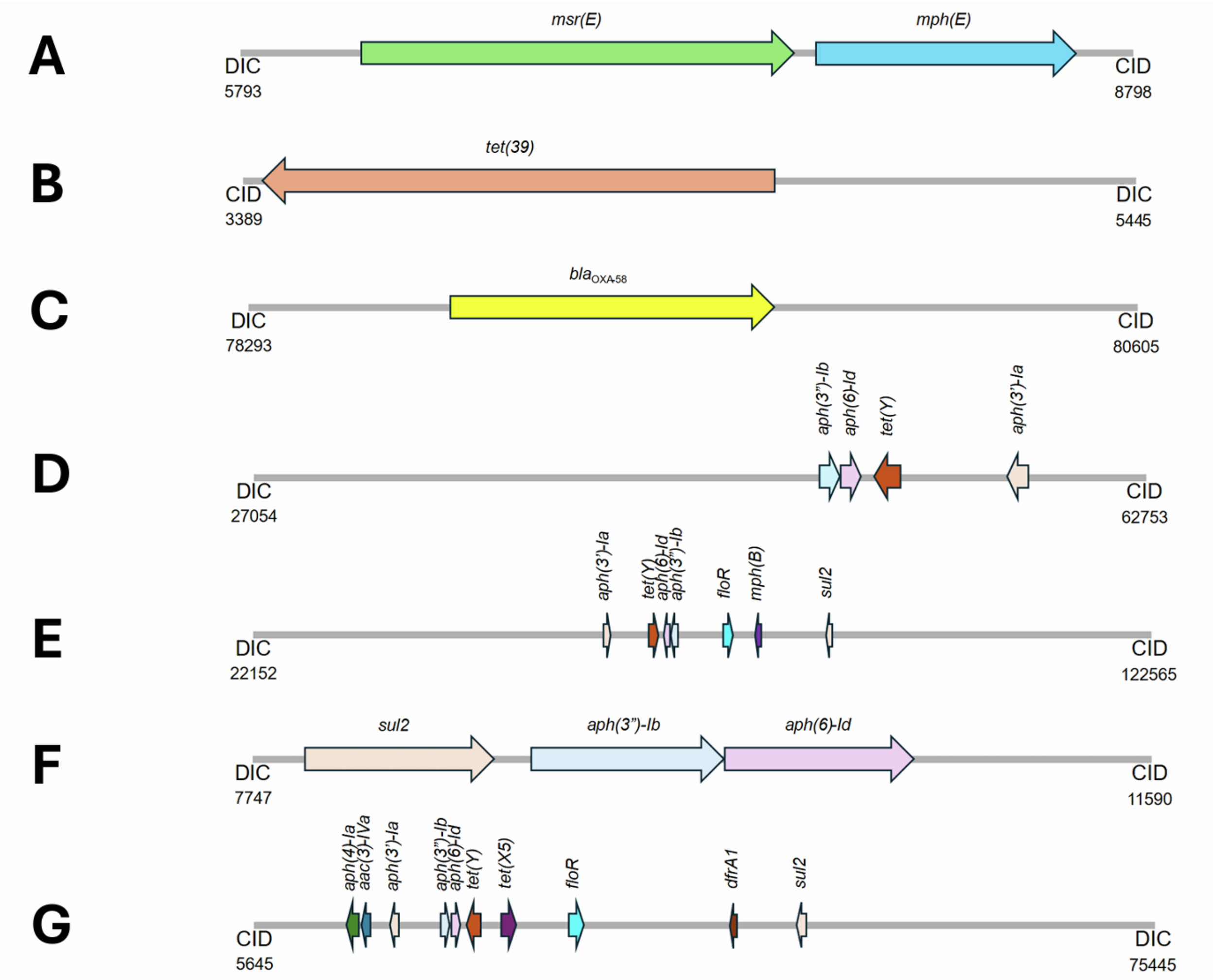

Supplementary Figure 4. ARGs within p*dif* modules. A. Macrolide resistance genes *msr(E)* and *mph(E).* **B.** Tetracycline resistance gene *tet(39).* **C.** Carbapenem resistance gene *bla*$_{OXA-58}$. **D.** Aminoglycoside resistance genes (*aph3-Ib*, *aph6-Id*, and *aph3-Ia*) and tetracycline resistance gene *tet(Y)*. **E.** Aminoglycoside resistance genes (*aph3-Ia*, *aph3-Ib*, and *aph6-Id*), macrolide resistance gene *mph(B)*, chloramphenicol resistance gene *floR*, sulfonamide resistance gene *sul2*, and tetracycline resistance gene *tet(Y).* **F.** Aminoglycoside resistance genes (*aph3-Ib* and *aph6-Id*). **G.** Aminoglycoside resistance genes (*aph4-Ia*, *aac3-IVa*, *aph3-Ia*, *aph3-Ib*, *aph6-Id*), tetracycline resistance genes (*tet(Y)* and *tet(X5)*), chloramphenicol resistance gene *floR*, trimethoprim resistance gene *dfrA*, and sulfonamide resistance gene *sul2.* The p*dif* sites were denoted as C|D and D|C.

Supplementary Figure 5.

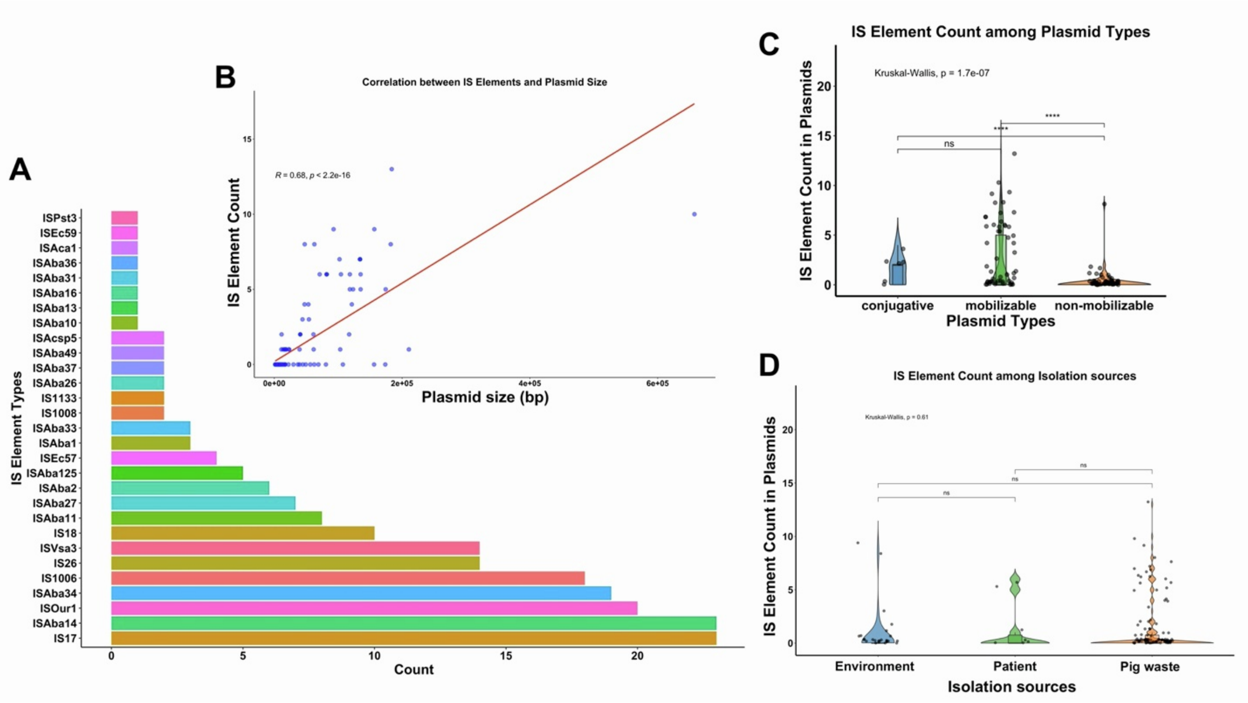

Supplementary Figure 5. Insertion sequence (IS) elements in plasmids. A. IS element types in plasmids. **B.** Correlation between IS element counts, and plasmid size using Spearman's rank correlation (R=0.68, *p*<2.2e-16). Each dot represents a plasmid. **C.** IS element distribution among plasmid types. Data are presented as median with interquartile range**. D.** IS element distribution among plasmid isolation sources. Kruskal-Wallis test was performed to compare the frequency of IS elements in C and D (*****p* <0.0001, ****p* <0.001). Data are presented as median with interquartile range.

Supplementary Figure 6.

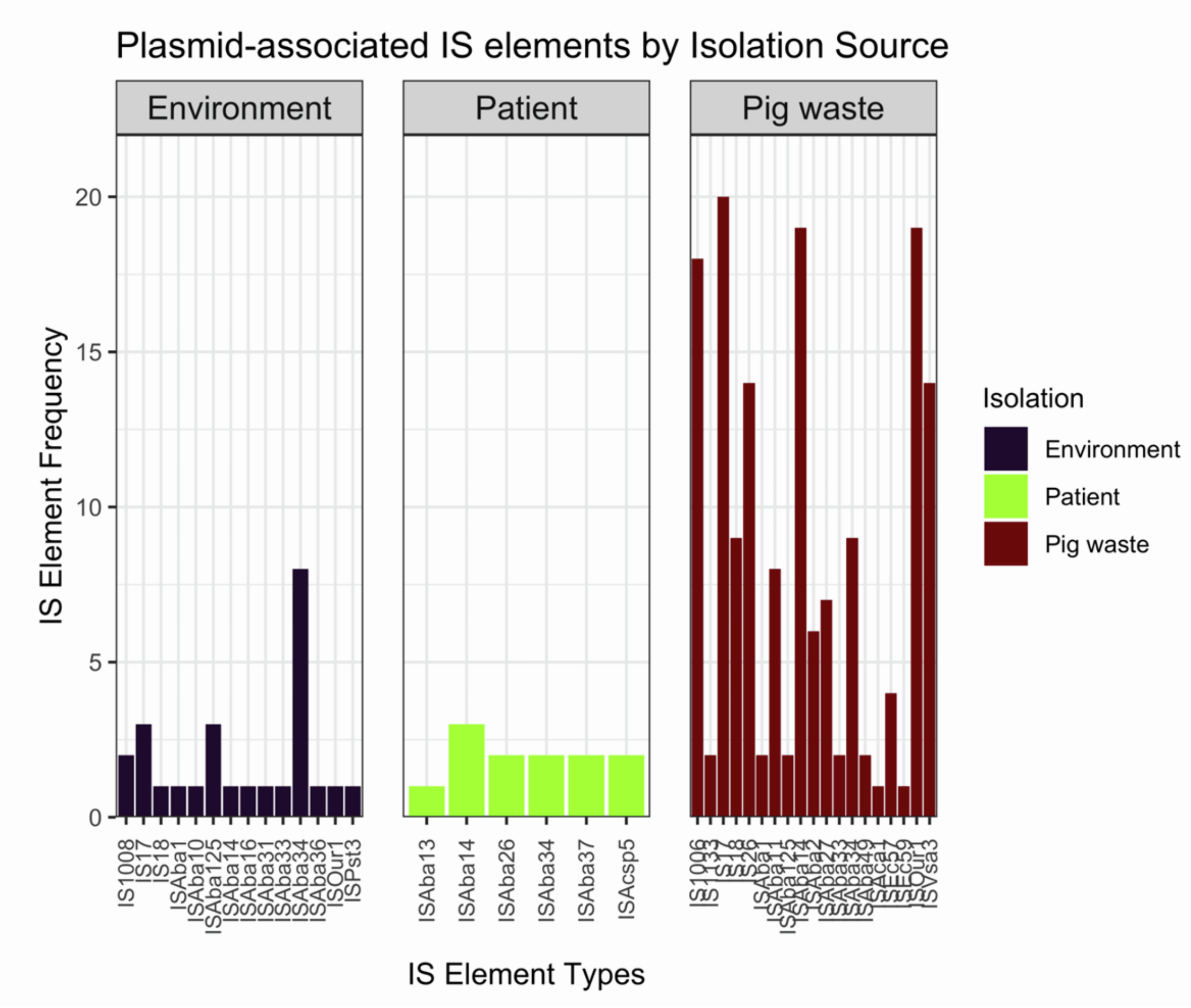

Supplementary Figure 6. Plasmid-associated IS elements. Unique IS element types among plasmids from different isolation sources.

Supplementary Figure 7.

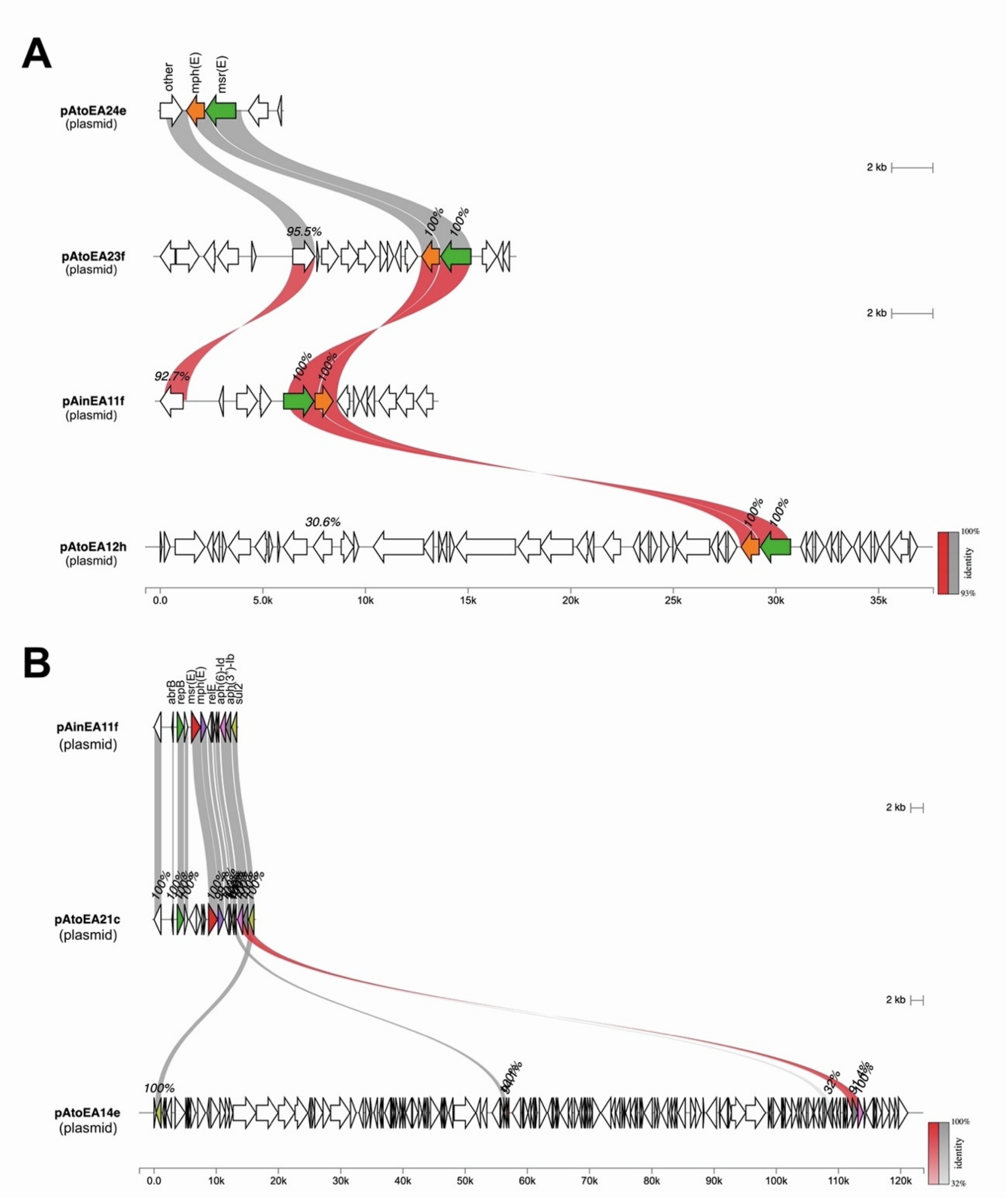

Supplementary Figure 7. Synteny analysis of plasmids originating from different pig-waste isolates. A. Macrolide resistance genes *msr(E)* and *mph(E)* among plasmids from pig-waste isolates. **B.** Aminoglycoside *aph3-Ib* and *aph6*-Id, macrolide *msr(E)* and *mph(E)*, and sulfonamide *sul2* resistance genes among plasmids from pig-waste isolate

Supplementary Figure 8.

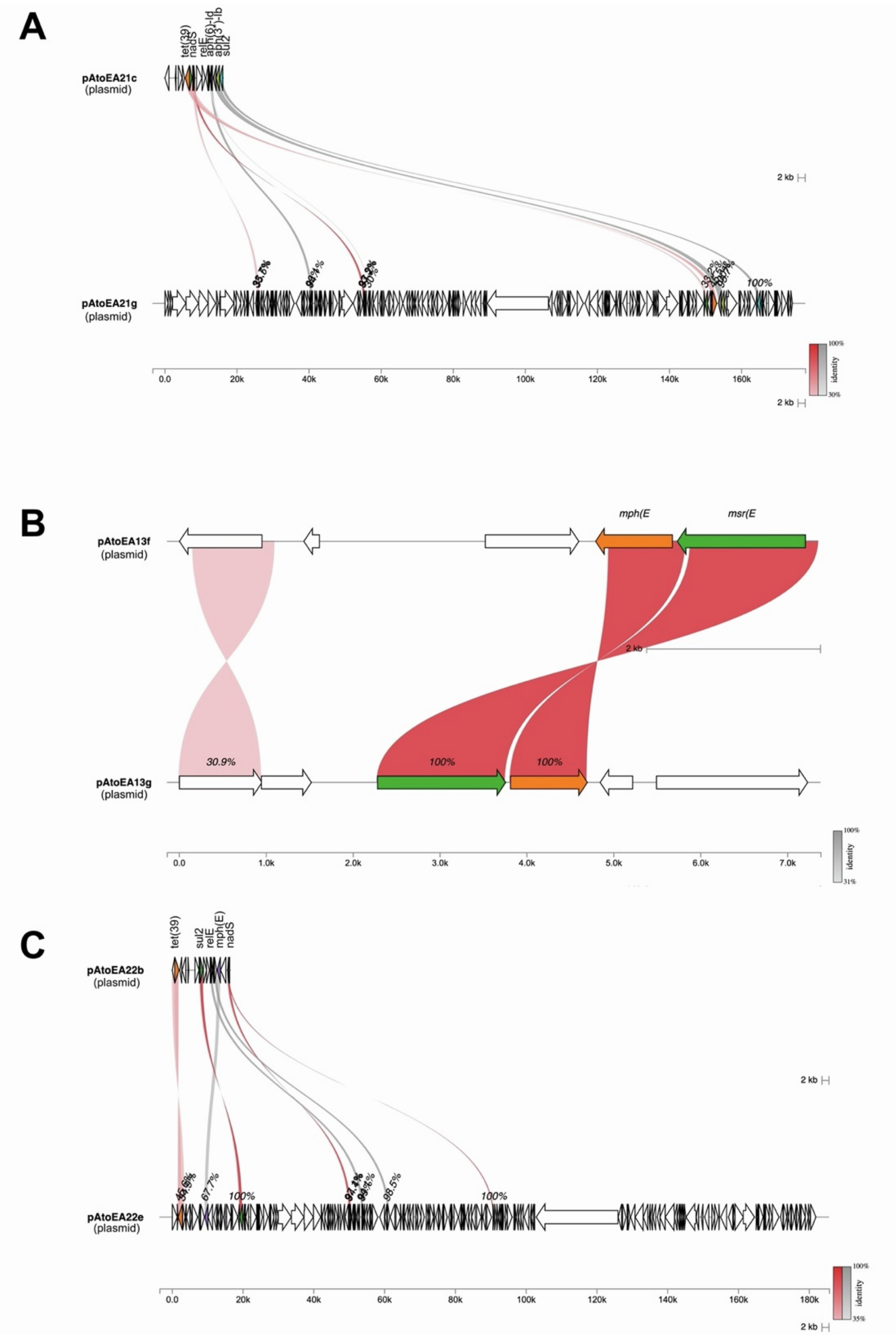

Supplementary Figure 8. Synteny analysis of co-residing plasmids from pig-waste isolates. A. Aminoglycoside (*aph3-Ib* and *aph6-Id*), sulfonamide *sul2*, and tetracycline *tet(39)* resistance genes in co-residing plasmids. **B.** Macrolide resistance genes *msr(E)* and *mph(E)* in co-residing plasmids. **C.** Macrolide *mph(E)*, sulfonamide *sul2*, and tetracycline *tet(39)* resistance genes in co-residing plasmids.

Supplementary Figure 9.

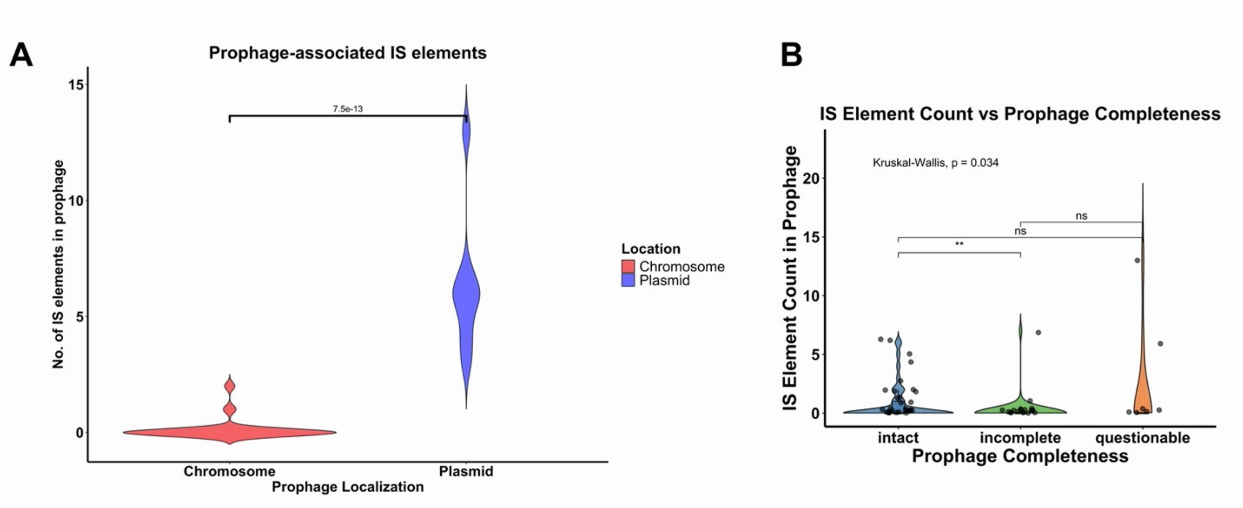

Supplementary Figure 9. IS element distribution in prophages. A. Distribution of IS elements in prophages in chromosomes and plasmids. Data are presented as median with interquartile range. **B.** Distribution of IS elements among different prophage completeness. Kruskal-Wallis test was performed to compare the frequency of IS elements (***p* <0.01). Data are presented as median with interquartile range.

Supplementary Table S2.

Plasmids harboring ARGs (n=42)		
**Plasmid data**	**Number of plasmids (n)**	**Percentage (%)**
**Isolation source:**	-	-
Environment (n=28)	1	3.57%
Pig waste (n=142)	40	28.17%
Patient (n=23)	1	4.35%
**Size:**	-	-
<20kb (n=142)	24	16.90%
20-<50kb (n=15)	5	33.33%
50-<100kb (n=14)	1	7.14%
100kb-<500kb (n=21)	12	57.14%
>500kb (n=1)	0	0%
**Mobility:**	-	-
Conjugative (n=7)	2	28.57%
Mobilizable (n=81)	24	29.63%
Non-mobilizable (n=105)	16	15.24%

Supplementary Table S3.

Pair No.	Total no. of plasmids harboring ARGs (n=42)	Plasmid number (n)	Percentage of co-localization (%)	Odds ratio	*p*-value (Fisher's exact test)
1	Plasmids with msr(E)	17	-	-	-
	Plasmids with mph(E)	17	-	-	-
	Plasmid with both ARGs	17	40.48%	Inf	3.93E-12
2	Plasmids with aph(6)-Id	17	-	-	-
	Plasmids with aph(3")-Ib	16	-	-	-
	Plasmid with both ARGs	16	38.10%	Inf	1.02E-10
3	Plasmids with aph(3")-Ia	9	-	-	-
	Plasmids with aph(3")-Ib	16	-	-	-
	Plasmid with both ARGs	9	21.43%	Inf	2.57E-05
4	Plasmids with tet(Y)	12	-	-	-
	Plasmids with sul2	16	-	-	-
	Plasmid with both ARGs	11	26.19%	47.65	1.04E-05
5	Plasmids with tet(Y)	12	-	-	-
	Plasmids with aph(3")-Ia	9	-	-	-
	Plasmid with both ARGs	9	21.43%	Inf	4.93E-07
6	Plasmids with blaOXA-58	8	-	-	-
	Plasmids with aph(3")-Ia	9	-	-	-
	Plasmid with both ARGs	7	16.67%	83.71	1.01E-05
7	Plasmids with aac(3)-IVa	4	-	-	-
	Plasmids with aph(4)-Ia	4	-	-	-
	Plasmid with both ARGs	4	9.52%	Inf	8.93E-06
8	Plasmids with tet(Y)	12	-	-	-
	Plasmids with tet(X5)	4	-	-	-
	Plasmid with both ARGs	4	9.52%	Inf	0.0044
9	Plasmids with floR	9	-	-	-
	Plasmids with mph(B)	7	-	-	-
	Plasmid with both ARGs	6	14.29%	51.92	0.0001
10	Plasmids with aph(6)-Id	17	-	-	-
	Plasmids with mph(B)	7	-	-	-
	Plasmid with both ARGs	7	16.67%	Inf	0.0007
11	Plasmids with sul2	16	-	-	-
	Plasmids with aph(3")-Ib	16	-	-	-
	Plasmid with both ARGs	14	33.33%	67.16	2.37E-07
12	Plasmids with blaOXA-58	8	-	-	-
	Plasmids with aph(3")-Ib	16	-	-	-
	Plasmid with both ARGs	7	16.67%	17.95	0.0026
13	Plasmids with sul2	17	-	-	-
	Plasmids with aph(6)-Id	17	-	-	-
	Plasmid with both ARGs	14	33.33%	45.13	1.25E-06

14	Plasmids with aph(3")-Ib	16	-	-	-
	Plasmids with aac(3)-IVa	4	-	-	-
	Plasmid with both ARGs	4	9.52%	Inf	0.0162
15	Plasmids with aph(3")-Ia	9	-	-	-
	Plasmids with aac(3)-IVa	4	-	-	-
	Plasmid with both ARGs	4	9.52%	Inf	0.0011
16	Plasmids with aph(3")-Ia	9	-	-	-
	Plasmids with sul2	16	-	-	-
	Plasmid with both ARGs	8	19.05%	22.82	0.0007
17	Plasmids with tet(Y)	12	-	-	-
	Plasmids with floR	9	-	-	-
	Plasmid with both ARGs	8	19.05%	48.93	3.38E-05
18	Plasmids with tet(Y)	12	-	-	-
	Plasmids with aph(3")-Ib	16	-	-	-
	Plasmid with both ARGs	11	26.19%	47.65	1.04E-05
19	Plasmids with tet(Y)	12	-	-	-
	Plasmids with aph(6)-Id	17	-	-	-
	Plasmid with both ARGs	11	26.19%	38.93	2.85E-05

Supplementary Table S5.

Host	plasmid	size	CDS	ARG	Completeness	Prophage GC%	Region Length (Kb)	# Total Proteins
A. towneri	pAtoEA12j	102107	105	+	incomplete	42.58	23.1	4
A. towneri	pAtoEA14e	121394	136	+	intact	39.53	34.8	18
A. towneri	pAtoEA21g	174353	169	+	intact	38.82	49.2	33
A. towneri					intact	42.72	46.2	30
A. towneri	pAtoEA22e	182229	175	+	intact	43.66	34.6	15
A. towneri	pAtoEA23i	183782	180	+	questionable	44.32	25.2	8
A. towneri	pAtoEA26d	81828	80	-	intact	37.64	31.5	21
A. towneri	pAtoEA28h	135132	163	+	questionable	43.04	17.2	20
A. guillouiae	pAguEC06e	44183	56	-	intact	36.87	18.1	21
